# A Treatise on Gonorrhœa and Syphilis

**Published:** 1864-10

**Authors:** 


A Treatise on Gonorrhœa and Syphilis. By Silas Durkee, M.D., Consulting Surgeon to the Boston City Hospital; Fellow of the Mass. Med. Soc.; &c., &c. Second Edition revised and enlarged. With eight colored Illustrations. Philadelphia: Lindsay & Blakiston. 1864.

This is a neatly printed volume of 467 pages, illustrated by eight very valuable colored plates. The style of the author is clear, concise, and pleasing. He has embraced in the work a consideration of the whole subject of gonorrhœal and syphilitic affections, both primary and remote. His opportunities for observation and practical experience in the treatment of these diseases appear to have been ample; to which he has added a thorough study of what has been observed and written by others. These advantages, added to a well-balanced and well-disciplined mind, have enabled the author to give us as good a practical treatise on these important topics as any with which we are acquainted. We recommend the work to our readers as one of positive merit, and worthy of the general patronage of the profession.

